# Small intestine choriocarcinoma with brain, lung and liver metastases in a male patient: a case report

**DOI:** 10.3389/fonc.2026.1874971

**Published:** 2026-07-27

**Authors:** Huamei Xu, Dayou Shi, Chuan Wang, Qingling Hua, Xiuling Wu

**Affiliations:** 1 Department of Oncology, The First Affiliated Hospital of Anhui Medical University, Hefei, China; 2 Oncology Department, Jiujiang City Key Laboratory of Cell Therapy, JiuJiang NO.1 People's Hospital, Jiujiang, China

**Keywords:** brain metastasis, liver metastasis, lung metastasis, small intestine choriocarcinoma, β-hCG

## Abstract

**Introduction:**

Extragonadal non-gestational choriocarcinama, especially in the gastrointestinal tract, is exceptionally rare. It often presents with metastatic disease and nonspecific symptoms, leading to diagnostic delays.

**Case report:**

Herein, we reported a 61-year-old male patient diagnosed with small intestine choriocarcinoma with metastases of brain, lung, liver and lymph nodes. This patient presented initially with black stools, heart palpitations and chest tightness. After diagnosis, the patient received chemotherapy with etoposide and cisplatin. The patient demonstrated a robust clinical and biochemical response to chemotherapy, with a significant reduction in β-HCG levels and increase of hemoglobin. Follow‐up imaging demonstrated a decreased size of pulmonary and liver metastases.

**Conclusions:**

Because of the complex nature of extragonadal non-gestational choriocarcinama, diagnosis is often delayed. The present case highlight the necessity for high clinical suspicion, and early initiation of treatment.

## Introduction

Choriocarcinoma is a rare but aggressive type of tumor which originates from trophoblast cells. The vast majority of choriocarcinomas occur in the context of pregnancy, particularly following molar pregnancy, abortion, or ectopic gestation. Non-pregnancy related choriocarcinoma accounts for less than 5% of cases and typically originates in gonads (1, 2). In male patient, the primary site of choriocarcinoma is usually found in testes. Several studies reported that extragonadal tumor sites can be detected in the mediastinum, retroperitoneum, lungs, brain, or gastrointestinal system (3). Symptoms of choriocarcinoma in men may be mild, like scrotal swelling and testicular mass. Nevertheless, patients may suffer with breath shortness, hemoptysis, abdominal pain, headaches etc. according to the primary or metastatic tumor sites. Extragonadal non-gestational choriocarcinama, especially in the gastrointestinal tract, is exceptionally rare, with very few cases previously reported in the literature (4, 5). When it occurs outside the gonads, it often presents with metastatic disease and nonspecific symptoms, leading to diagnostic delays. Herein, we report a diagnostic journey in a 61 years old male patient with choriocarcinoma originating in the small intestine. This case was initially diagnosed with gastrointestinal stromal tumor or lung adenocarcinoma, however ultimately diagnosed with choriocarcinoma through integrated imaging, serum biomarker, and comprehensive histology and immunohistochemistry.

## Case report

**Figure 1:**
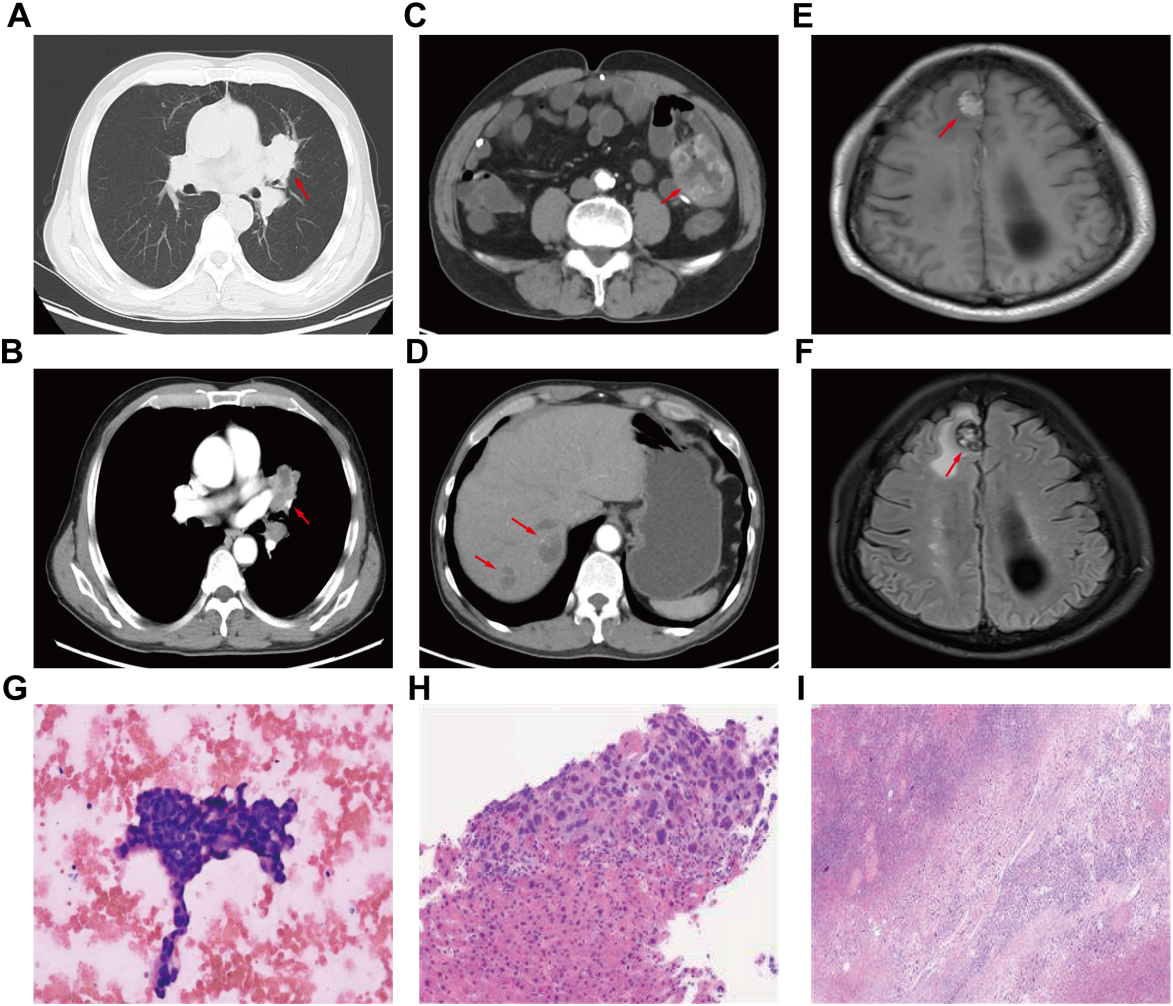
Basic clinical characteristics of the patient. **(A, B)** Mass in the left upper lobe of lung and enlarged lymph nodes in the left hilum. **(C)** Lesion in the jejunum at the left upper abdomen. D Lesions in the liver. **(E, F)** Mass in the right frontal lobe accompanied by hemorrhage. **(G)** Typical picture of H&E staining of cells from the mass in lung and enlarged lymph nodes in left hilum. **(H)** Typical picture of H&E staining of tumor tissue from liver. **(I)** Typical picture of H&E staining of tumor tissue from small intestine.

**Figure 2:**
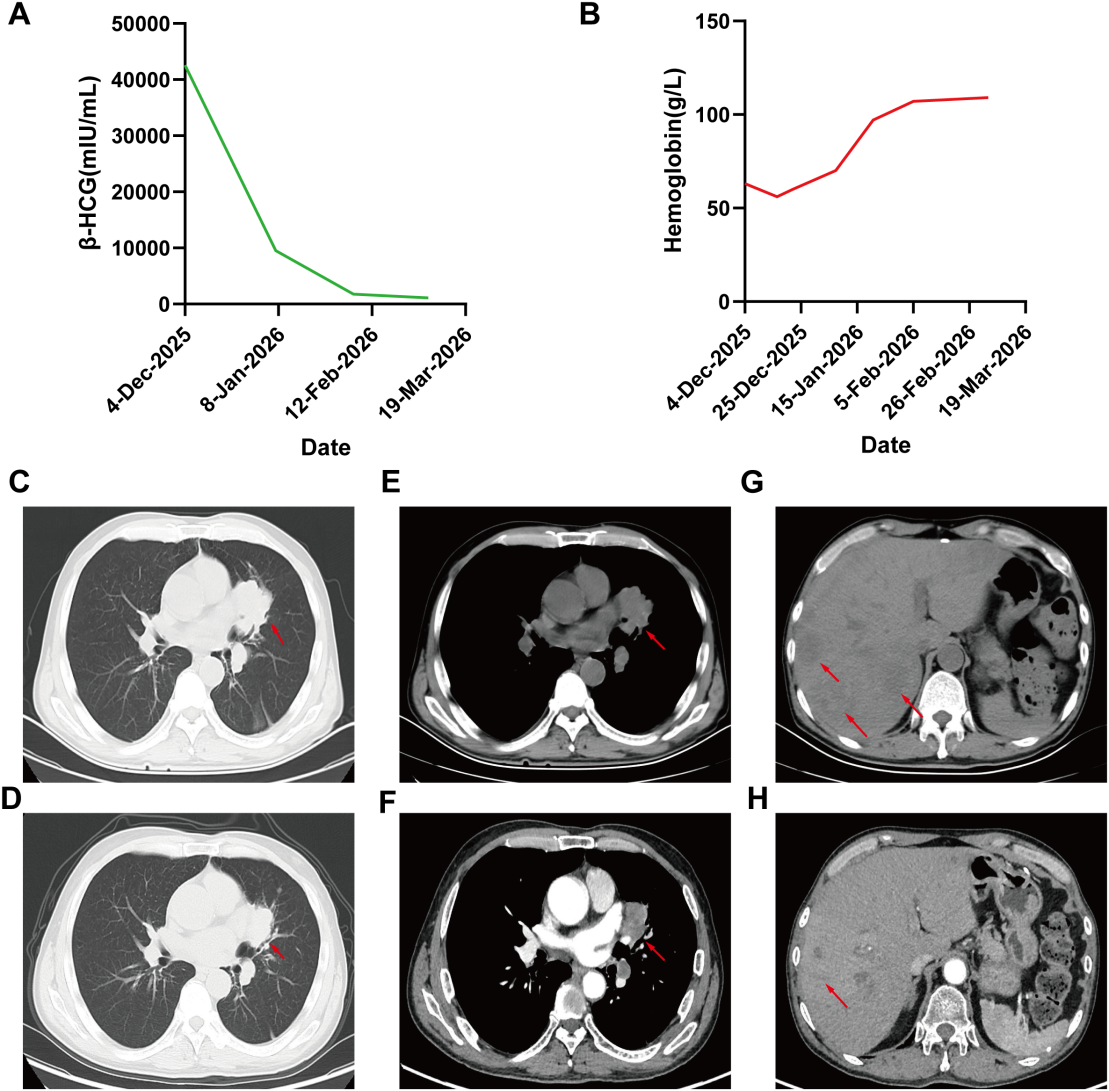
Follow up tests of the patient before and after treatments. **(A)** Change of β-HCG levels before and after chemotherapies. **(B)** Change of hemoglobin levels before and after chemotherapies. **(C-F)** Changes of mass in the left upper lobe of lung and enlarged lymph nodes in the left hilum after chemotherapies. **(G, H)** Changes of tumors in liver after chemotherapies.

A 61-year-old male presented to our hospital complaining of black stools for 4 months, heart palpitations and chest tightness after activity for 2 weeks. His associated symptoms included 2.5kg weight loss and decreased appetite. His medical history included hypertension and he had undergone ventriculoperitoneal shunt for hydrocephalus 15 years ago. The results of regular examinations were as follows: normal vital signs, fecal occult blood test (++), hemoglobin 46 g/L (range:120–160 g/L). Liver and kidney function, electrolytes and blood glucose were normal. A computed tomography (CT) scan of chest, abdomen, and pelvis revealed a mass in the left upper lobe of lung, enlarged lymph nodes in the left hilum and mediastinum, a lesion in the jejunum at the left upper abdomen (radiological interpretation was suggestive of high-risk gastrointestinal stromal tumor (GIST) accompanied by mesenteric lymphadenopathy), and lesions of low density in the liver (indicating metastatic tumors) (**Figures 1A–D**). Cranial magnetic resonance imaging (MRI) demonstrated a mass in the right frontal lobe, consistent with a metastatic tumor accompanied by hemorrhage (**Figures 1E, F**). Tumor markers were as follows: normal range of carcinoembryonic antigen (CEA), alpha-fetoprotein (AFP) and carbohydrate antigen 19-9 (CA19-9); neuron-specific enolase (NSE) 22.10 ng/ml (range:2–20 ng/ml); cytokeratin 19 fragment (CYFRA21-1) 48.56 ng/ml (range:0-3.3 ng/ml); serum ferritin (SF) 1688.6 ng/ml (range:30–406 ng/ml). Considering the presence of anemia and melena of this patient, gastroscopy was performed, which only showed chronic non-atrophic gastritis (active stage). Colonoscopy revealed no obvious abnormalities in the terminal ileum, colon and rectum. No neoplasms were identified in bronchus and its branches by bronchoscopy. Endobronchial ultrasound revealed enlarged lymph nodes and mass. Biopsy of the nodes and mass showed a small number of mildly atypical cells (**Figure 1G**). The immunohistochemistry tests of these cells:CK(+), TTF-1(-), NapsinA(-), CK7(+), P63(-), P40(-), Ki-67(about 75%+), syn(-), CD56(-), LCA(+). The pathologist suggested that adenocarcinoma should be considered based on the findings of Hematoxylin and eosin (H&E) staining and immunohistochemical analyses. However, the immunohistochemical findings did not support a diagnosis of primary pulmonary adenocarcinoma, and metastatic adenocarcinoma from an unknown primary site remained a differential consideration. Liver biopsy detected malignant tumor with necrosis (**Figure 1H**). The immunohistochemistry tests of liver biopsy:CK(+), Ki-67(about 75%+), Vim(-), CD117(-), CD34(-), CK7(partial +), TTF-1(-), GS(-), CK5/6(-), P40(-), CK19(+). On November 18, 2025, the patient underwent laparoscopic exploration and partial small bowel resection. Postoperative pathology revealed: (partial small bowel) malignant tumor with necrosis, invading the subserosal layer; tumor size: 11.5 cm×6.6 cm×1.3 cm, with extensive hemorrhage and necrosis, involving the full thickness of the tissue; absence of neural invasion; absence of lymphovascular invasion; both resection margins and circumferential margins were free of tumor (**Figure 1I**). Immunohistochemistry showed: CK(+), Ki-67 (>80%+), SALL4 (partial+), HCG-B (partial+), CD117(-), DOG-1(-), CD34(-), S-100(-), CDX-2(-), CK20(-), Glypican-3(-), INI-1(-), BRG-1(-), AFP(-), PLAP(-). Pathological diagnosis: low-grade adenocarcinoma of the small bowel is considered, however, choriocarcinoma was also on the list. Later, the serum beta-human chorionic gonadotropin (β-HCG) was tested and the result was 42573.53 mIU/mL (range:0-5.3 mIU/mL). No obvious abnormalities were detected in his testes. Considering the super high β-HCG and characteristics of imaging and pathology, the patient was diagnosed with small intestine choriocarcinoma with metastases of brain, lung, liver and lymph nodes. After diagnosis, the patient received chemotherapy with etoposide 60mg/m^2^×5 days and cisplatin 75mg/m^2^ every 3 weeks (EP). The patient received adequate hydration and diuresis throughout chemotherapy. During chemotherapy, the patient experienced no severe adverse reactions, but only mild leukopenia and anorexia. The patient showed a significant response to chemotherapy, with a significant reduction in β-HCG levels (**Figure 2A**). Hemoglobin increased rapidly among the period time of treatments (**Figure 2B**). Regular contrast-enhanced CT of the chest, abdomen and pelvis, and cranial MRI were performed after every 2 cycles of chemotherapy. Follow‐up images demonstrated a decreased size of pulmonary and liver metastases (**Figures 2C–H**). He continues to receive treatment and undergo regular monitoring of tumor markers and imaging to assess disease progression. Until 2026 June, his tumor maintains sustained remission with good general conditions.

## Discussion

Choriocarcinoma is incredibly uncommon in male. Testicular choriocarcinoma makes up only 0.01%–0.02% of malignancies in men and less than 1% (0.19%) of testicular germ cell cancers (6). Pathology is the diagnostic gold standard of choriocarcinoma. The histopathologic characteristics of typical choriocarcinoma include columns of trophoblastic cells without villous structures. Blood vessels and muscle often be invaded. Furthermore, necrosis and hemorrhage often can be detected in tumor tissue either (7). Serum β-HCG plays a key role in both the diagnosis and monitor of choriocarcinoma. According to previous literature, high β-HCG (more than 1000 mlU/mL) is vital evidence for choriocarcinoma diagnosis. Moreover, the level of β-HCG can be used to estimate the burden of tumor (8, 9). Thus, levels of β-HCG should be monitored over the period time of treatment to assess the therapeutic effect. The level of the present case was 42573.53mIU/mL (range:0-5.3). According to the International Germ Cell Carcinoma Collaborative Group, patient’s prognosis will be worse if their β-HCG were more than 50,000 mIU/mL.

Before the diagnosis of small intestine choriocarcinoma, several diseases should be included in the differential diagnosis. 1. Small bowel GIST: The initial suspicion of GIST was reported by radiologist based on the large intestinal mass. However, GIST typically presents as an exophytic hypervascular mass without extremely high serum β-HCG. Moreover, the immunohistochemistry of GIST is positive for CD117 and DOG-1, which were both negative in our case. In addition, GIST rarely causes extensive multi-organ metastasis and extreme anemia due to massive hemorrhage, which excludes this diagnosis. 2. Small bowel adenocarcinoma: Small bowel adenocarcinoma usually manifests as intestinal wall thickening, annular stricture, and mild homogeneous enhancement, without extensive massive hemorrhage and necrosis. It is accompanied by elevated CEA/CA19–9 rather than extremely high serum β-HCG, and positive gastrointestinal markers (CDX2, CK20), which are inconsistent with our patient’s findings. 3. Small bowel lymphoma: Lymphoma typically presents with diffuse bowel wall thickening and homogeneous mild enhancement, with rare massive hemorrhage and necrosis. It is immunohistochemically positive for lymphocyte markers and negative for β-HCG, which does not match the present case. 4. Metastatic tumors (e.g., melanoma and lung cancer etc.): These metastatic lesions often present as hypervascular intestinal masses, but they are not associated with significantly elevated serum β-HCG, and have distinct primary tumor imaging and pathological features, which can be distinguished from choriocarcinoma. 5. Benign small bowel lesions with hemorrhage/mass: Crohn’s disease or anticoagulation-related hematoma may cause intestinal wall thickening and bleeding, but no malignant proliferative mass or metastasis is observed, and no abnormal elevation of tumor markers exists, which can be completely excluded.

It is worth noting that adenocarcinoma was firstly considered by pathologist based on the tissue of small bowel lesion. Previous literature reported case of small bowel adenocarcinoma with choriocarcinoma differentiation (10). We believed that this disease also should be included in our differential diagnoses. Small bowel adenocarcinoma with choriocarcinoma differentiation is a mixed malignant tumor dominated by adenocarcinoma components, with partial secondary choriocarcinomatous differentiation. Its immunohistochemistry is mainly positive for gastrointestinal adenocarcinoma markers (CDX2, CK20), with only focal and weak β-HCG expression. In contrast, primary small bowel choriocarcinoma is a pure trophoblastic malignant tumor characterized by massive hemorrhage and necrosis, with diffuse high Ki-67 proliferation, focal SALL4 and β-HCG positivity, and negative gastrointestinal adenocarcinoma markers (CDX2, CK20, Glypican-3), which is consistent with our patient’s immunohistochemical results. The initial tentative pathological consideration of adenocarcinoma in our case was attributed to the atypical morphological changes caused by extensive tumor necrosis, rather than true adenocarcinoma components. Adenocarcinoma with choriocarcinoma differentiation usually presents with chronic intestinal obstruction, abdominal pain, and mild gastrointestinal bleeding, with slightly elevated or normal serum β-HCG. Primary small bowel choriocarcinoma is characterized by severe melena, refractory severe anemia, extensive tumor hemorrhage and necrosis, accompanied by extremely high serum β-HCG (consistent with 42573.53 mIU/mL in our case), and is more prone to early distant multi-organ metastasis. Small bowel adenocarcinoma with choriocarcinoma differentiation follows the treatment principle of intestinal adenocarcinoma, mainly relying on surgical resection combined with gastrointestinal tumor chemotherapy regimens (e.g., FOLFOX), with poor sensitivity to trophoblastic tumor chemotherapy. In contrast, primary small bowel choriocarcinoma is managed as a high-grade malignant trophoblastic tumor. The standard first-line EP chemotherapy regimen is highly sensitive, with rapid tumor regression and significant β-HCG reduction. Our patient’s dramatic response to EP chemotherapy further excludes the diagnosis of adenocarcinoma with choriocarcinoma differentiation and confirms the final diagnosis of primary small bowel choriocarcinoma.

In this case, colonoscopy revealed no obvious abnormalities in the terminal ileum, colon and rectum. Jejunum, the lesion site of our patient, is beyond the reach of routine upper and lower endoscopy. In addition, this choriocarcinoma grows as an intramural/submucosal mass without prominent mucosal ulceration or exophytic polypoid lesions on the luminal surface. These two limitations render conventional endoscopy unable to detect jejunal intramural malignant lesions, consistent with the negative endoscopic findings in this case. Moreover, the assessment of reproductive system and HCG testing were absent for the initial examinations, resulting in diagnostic delays. Early and accurate diagnosis of choriocarcinoma is critical, as delayed diagnosis is associated with increased mortality, prolonged hospital stays, and higher medical costs. Therefore, initial measurement of β-HCG is indispensable. Prevention of tumor rupture and hemorrhage and optimizing general condition of patient are key to effective therapy. Overall, early recognition, prompt intervention, and precise diagnosis are crucial for improvements of patient prognosis.

Metastases of choriocarcinoma often can be found in the lungs (80%), followed by the vagina (30%), pelvis (20%), liver (10%), and brain (10%) (3, 11). This patient has tumors in sites including small intestine, brain, lung and liver. It is difficult to distinguish the original site from metastatic sites for this patient. Considering the big size of tumor in small intestine and his first symptom (black stools), this patient was diagnosed with small intestine choriocarcinoma with metastases of brain, lung, liver and lymph nodes.

Small intestinal choriocarcinoma is extremely rare, with only a small number of case reports documented in previous literature (12–15). Herein, we briefly summarize previously reported cases and compare them with the present case (**Table 1**). Early therapy is important to prognosis of choriocarcinoma patient. Appropriate chemotherapy has possibilities to cure choriocarcinoma patients even with metastases, because choriocarcinomas are highly susceptible to chemotherapy. It has been reported that survival rate of choriocarcinoma patients undergone surgery was only 19%, however chemotherapy can lift the survival rate to more than 90% (16). Cisplatin based chemotherapy is the gold standard treatment for this disease. Available chemotherapy regimens consist of etoposide and cisplatin (EP), bleomycin, etoposide and cisplatin (BEP), and vinblastine or etoposide with isophosphamide and cisplatin (VIP) (17). In recent years, immunotherapy (i.e., immune checkpoint blockade) combined with chemotherapy show more effectiveness in some patients with resistance to chemotherapy alone (18–20).

**Table 1:** Comparisons of the present case and previously reported cases.

Study	Year	Gender	Age	Primary Symptoms	Primary site	Metastatic Sites	Treatment	Clinical Outcome
Engel et al. (12)	1979	Male	30	Abdominal pain, melena, vomiting	duodenum	Lung, liver, retroperitoneum	Cisplatin + vinblastine + bleomycin	Died of irreversible respiratory failure
Harada et al. (13)	1991	Female	48	Postprandial upper abdominal pain, vomiting	jejunum	No distant metastasis	Partial jejunectomy only	Postoperative serum HCG normalized, no follow-up death record
Ravi et al. (15)	1997	Female	22	High fever, abdominal pain, intestinal perforation and obstruction	Jejunum and ileum	Liver, spleen	Emergency small bowel resection; planned chemotherapy not fully implemented	Died on postoperative day 8 from massive gastrointestinal hemorrhage
Iyomasa et al. (14)	2003	Male	45	Melena, severe anemia, abdominal perforation	Jejunum	Liver	Jejunal resection + bleomycin+etoposide+cisplatin (BEP)	Died 5 months postoperatively due to progressive liver metastases
Our case	2026	Male	61	Melena, exertional palpitations, chest tightness, weight loss, poor appetite	Jejunum	lung, liver,brain, mediastinal/hilar lymph node metastases	Laparoscopic partial small bowel resection + EP chemotherapy (etoposide + cisplatin)	Sharp decline of HCG, evident shrinkage of metastases

Serial follow-up imaging examinations confirmed continuous shrinkage of pulmonary, hepatic, and lymph node metastatic lesions, and stable metastatic lesion in the brain. No new metastatic lesions or disease progression were observed during the follow-up period. Combined with the continuous decline of serum β-HCG and the recovery of hemoglobin, the follow-up imaging findings further verified the excellent therapeutic effect of chemotherapy on this rare small bowel choriocarcinoma with multi-organ metastasis.

## Conclusion

Choriocarcinoma in male is a rare, aggressive tumor with nonspecific symptoms. Due to the complex nature of this disease, diagnosis is often delayed. The present case highlight the necessity for high clinical suspicion, and early initiation of treatment.

## Data Availability

The original contributions presented in the study are included in the article/supplementary material, Further inquiries can be directed to the corresponding authors.
